# Pleuropneumonia Revealing Angioimmunoblastic T-cell Lymphoma

**DOI:** 10.7759/cureus.80268

**Published:** 2025-03-08

**Authors:** Zineb Salhi, Afaf Thouil, Mohamed Lakhal, Sara Gartini, Meriem Rhazari, Ikram Sadki, Nassira Karrich, Hatim Kouismi

**Affiliations:** 1 Department of Respiratory Diseases, Centre Hospitalier Universitaire (CHU) Mohammed VI, Faculty of Medicine and Pharmacy, Mohammed I University, Oujda, MAR; 2 Department of Respiratory Diseases, Laboratory of Research and Medical Sciences, Faculty of Medicine and Pharmacy of Oujda, Mohammed VI University Hospital, Mohammed I University, Oujda, MAR; 3 Department of Internal Medicine, Laboratory of Immunohematology and Cellular Therapy, Faculty of Medicine and Pharmacy of Oujda, Mohammed I University, Oujda, MAR; 4 Department of Anatomopathology, Faculty of Medicine and Pharmacy of Oujda, Mohammed I University, Oujda, MAR

**Keywords:** adenopathy, biopsy, chemotherapy, lymphoma, node, pleuropneumonia

## Abstract

Angioimmunoblastic T-cell lymphoma (AITL) is a rare form of lymphoma that presents with a wide range of symptoms, including multiple lymphadenopathies, systemic symptoms, splenomegaly, hepatomegaly, and skin rashes. Due to its atypical presentation, diagnosis can be delayed, and confirmation relies on lymph node biopsy. While no specific risk factors or etiological agents have been identified, early diagnosis and appropriate treatment, such as polychemotherapy, can lead to a favorable prognosis. This case report describes an instance of AITL diagnosed following the onset of pleuropneumopathy, emphasizing the importance of considering lymphoma in the differential diagnosis of pleuropulmonary conditions.

## Introduction

Angioimmunoblastic T-cell lymphoma (AITL) is an aggressive subtype of peripheral T-cell lymphoma (PTCL) [1]. Due to diagnostic challenges, its incidence is likely underestimated. Diagnosis is typically confirmed through lymph node biopsy, identifying a clonal T-cell population as a key diagnostic criterion [2]. While AITL primarily affects lymph nodes, it may also involve the spleen, bone marrow, and liver. However, pleuropulmonary involvement remains exceptionally rare [2].

First described in the 1970s, AITL was initially considered a reactive hyperplasia before being recognized as a distinct lymphoproliferative disorder. It has since been linked to immune dysregulation, polyclonal hypergammaglobulinemia, and an association with Epstein-Barr virus, which may play a role in its pathogenesis. Recent advances have also identified key genetic mutations, such as Ras homolog family member A, and epigenetic changes affecting follicular helper T cells, which contribute to the development of the disease. These findings have led to exploring new therapeutic strategies, including histone deacetylase inhibitors and monoclonal antibodies, alongside conventional chemotherapy. Emerging immunotherapeutic approaches also hold promise for improving outcomes in this challenging disease. We report the case of a patient admitted to our pulmonology department and diagnosed with AITL, revealed by pleuropneumonia.

## Case presentation

A 63-year-old female patient, previously diagnosed with tuberculosis of the lymph node one year ago and treated with antituberculosis treatment based on rifampicin, isoniazid, ethambutol, and pyrazinamide therapy for three months before the treatment was discontinued, presented with worsening symptoms over the past four months. The primary complaints included a dry cough, exertional dyspnea, and diffuse chest pain, all occurring in the context of a general deterioration in health, characterized by profound asthenia and a weight loss of 7 kg over four months.

On general examination, the patient was classified with a performance score of 4 and exhibited respiratory instability, with an oxygen saturation (SaO_2_) of 89%-91% on room air. Oxygen therapy at a flow rate of 2 L/minute was initiated, improving saturation to 94%.

The pulmonary examination revealed an effusion syndrome on the left side, while the abdominal examination showed painless hepatomegaly with a liver span of 14 cm and splenomegaly. Additionally, a 2 cm × 2 cm nonfistulized left axillary adenopathy was noted, fixed to the deep plane but mobile relative to the superficial plane and without inflammatory signs. The rest of the physical examination was unremarkable.

**Figure 1:**
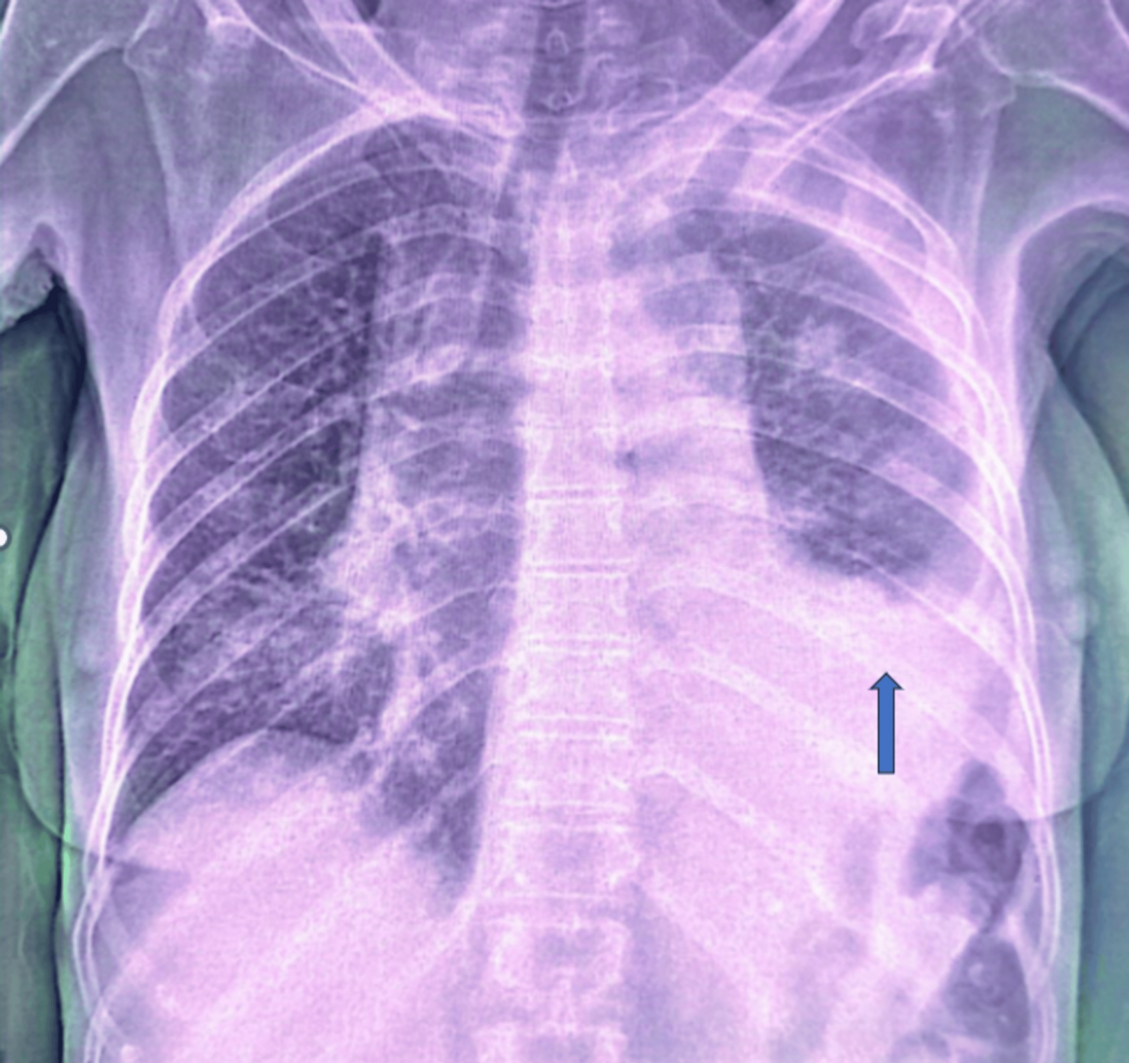
Front view of the chest X-ray with moderate left-sided pleurisy (blue arrow)

**Figure 2:**
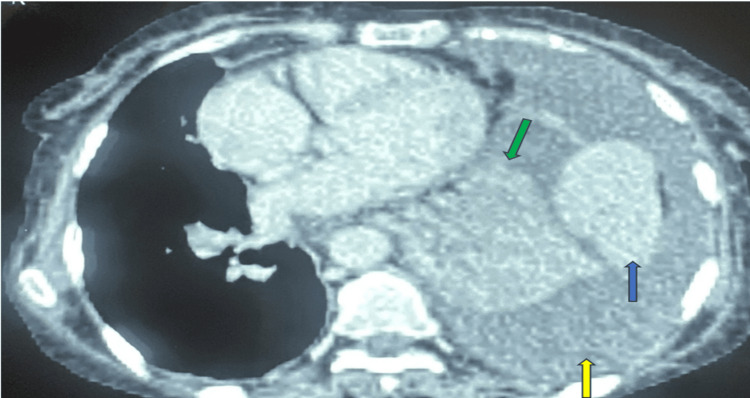
Axial section of the thoracic CT scan showing a focus of parenchymal consolidation in the left upper lobe, associated with nodular consolidations (blue arrow), a moderately abundant pleural effusion (yellow arrow), and a collapsed lung (green arrow) CT: computed tomography

Imaging studies provided further insights. A chest X-ray (Figure 1) showed moderate left-sided pleurisy, while a chest CT scan (Figure 2) highlighted a heterogeneous triangular consolidation in the left upper lung parenchyma. This lesion was characterized by an air bronchogram and nodular areas of consolidation. The CT scan also revealed bilateral bronchopneumonic infiltrates, which strongly suggested a lymphomatous etiology.

As part of the etiological diagnostic process, the patient underwent several investigations. A complete blood count revealed a leukocyte count of 7,980 cells/mm³, with 4,670 neutrophils/mm³. The patient also presented with microcytic hypochromic anemia, evidenced by a hemoglobin level of 9 g/dL, while lymphocyte and platelet counts were within normal ranges. Liver function tests were normal, although the lactate dehydrogenase (LDH) level was elevated at 736 U/L, and renal function tests showed no abnormalities.

Analysis of the right-side pleural fluid obtained by thoracentesis indicated an exudate with elevated fluid protein, whereas serum protein, fluid LDH, and serum LDH were within the normal range (Table 1).

**Table 1:** Laboratory results of the right-side pleural fluid obtained by thoracentesis

Laboratory test	Value	Reference range
Fluid protein (g/L)	48	<20
Serum protein (g/L)	73	60-80
Serum lactate dehydrogenase (U/L)	210	140-280
Fluid lactate dehydrogenase (U/L)	143	<200

**Figure 3:**
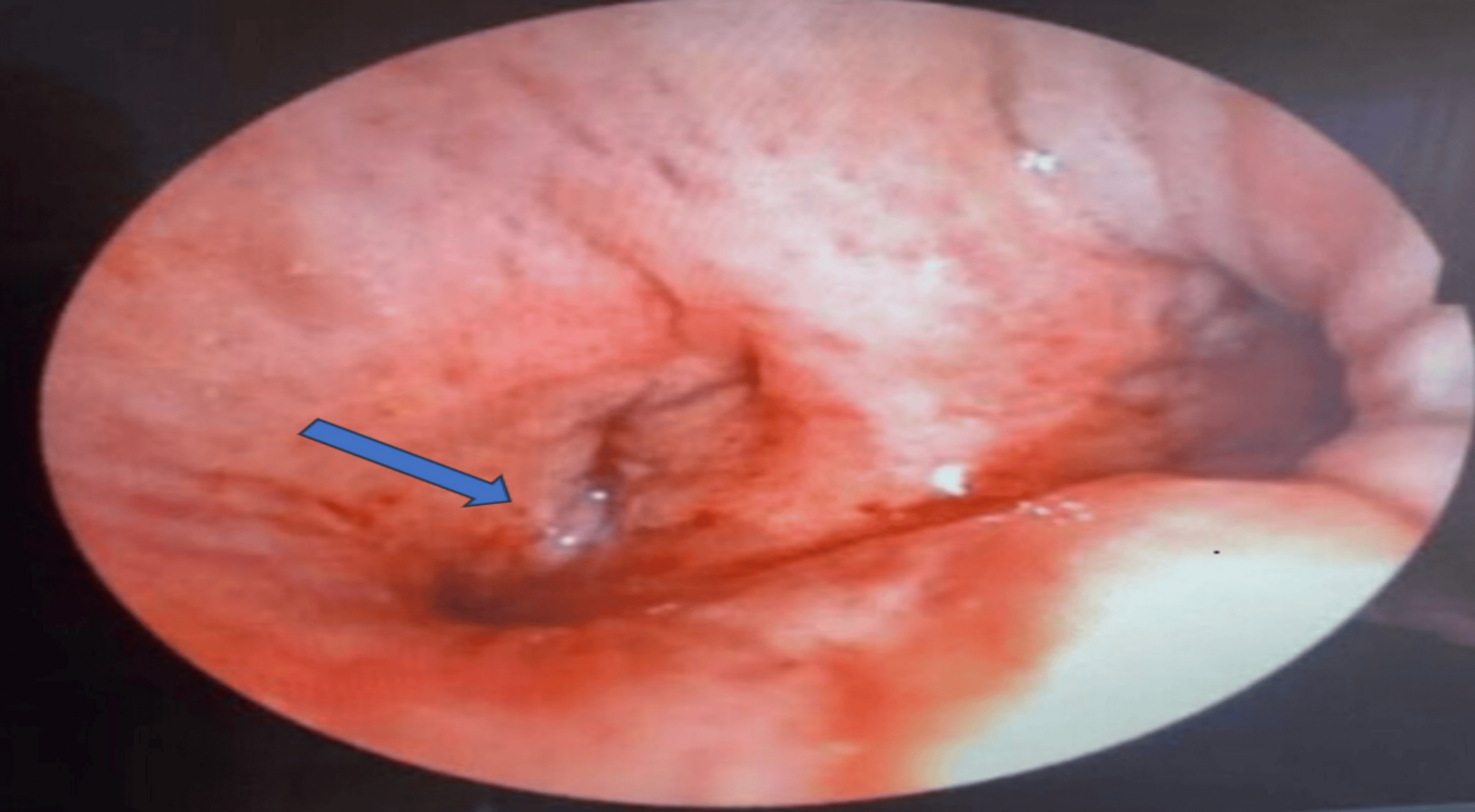
Bronchoscopy image showing infiltrative stenosis of the left upper lobar bronchus (blue arrow)

Further investigations included two blind pleural biopsies, both of which were inconclusive. Flexible bronchoscopy revealed infiltrative stenosis of the left upper lobar bronchus and thickening located between the lingula and the culmen (Figure 3). Bronchial biopsies indicated nonspecific chronic inflammatory bronchitis. Additionally, bronchial aspirates were negative for neoplastic cells, and the Xpert MTB/RIF assay (Cepheid Inc., Sunnyvale, CA) for acid-fast bacilli yielded negative results.

A cerebral, thoracic, abdominal, and pelvic computed tomography (CT) scan revealed multiple adenopathies above and below the diaphragm, hepatosplenomegaly, and mild ascites. Subsequently, a biopsy of the axillary adenopathy was performed. Histopathological and immunohistochemical studies confirmed the diagnosis of AITL.

**Figure 4:**
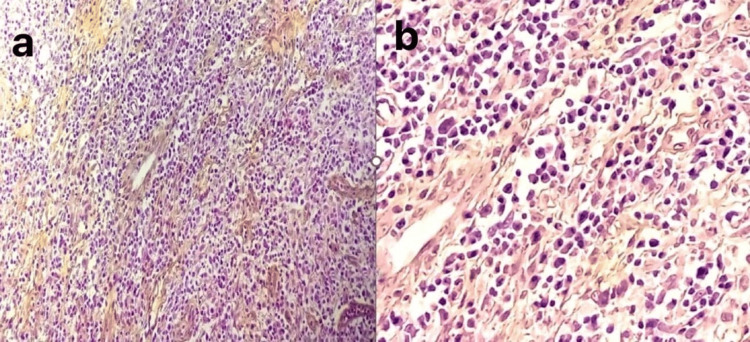
(a) Prominent high endothelial venules were visible. (b) Hematoxylin-eosin-saffron staining showing a diffuse infiltrate of small- to medium-sized lymphoid cells with scattered admixed plasma cells

Histological examination showed that the lymph node parenchyma was predominantly effaced and replaced by a lymphoid proliferation arranged in diffuse sheets of medium-sized cells with irregularly contoured, hyperchromatic, round nuclei and clear cytoplasm. These cells exhibited an immunoblastic appearance in some areas, characterized by increased cell size. The tumor cells were embedded in a polymorphic inflammatory background of lymphocytes, plasma cells, neutrophils, eosinophils, and histiocytes, with associated hyperplasia of vascular structures. Peripheral infiltration of the adipose tissue by tumor cells was also noted (Figure 4).

**Figure 5:**
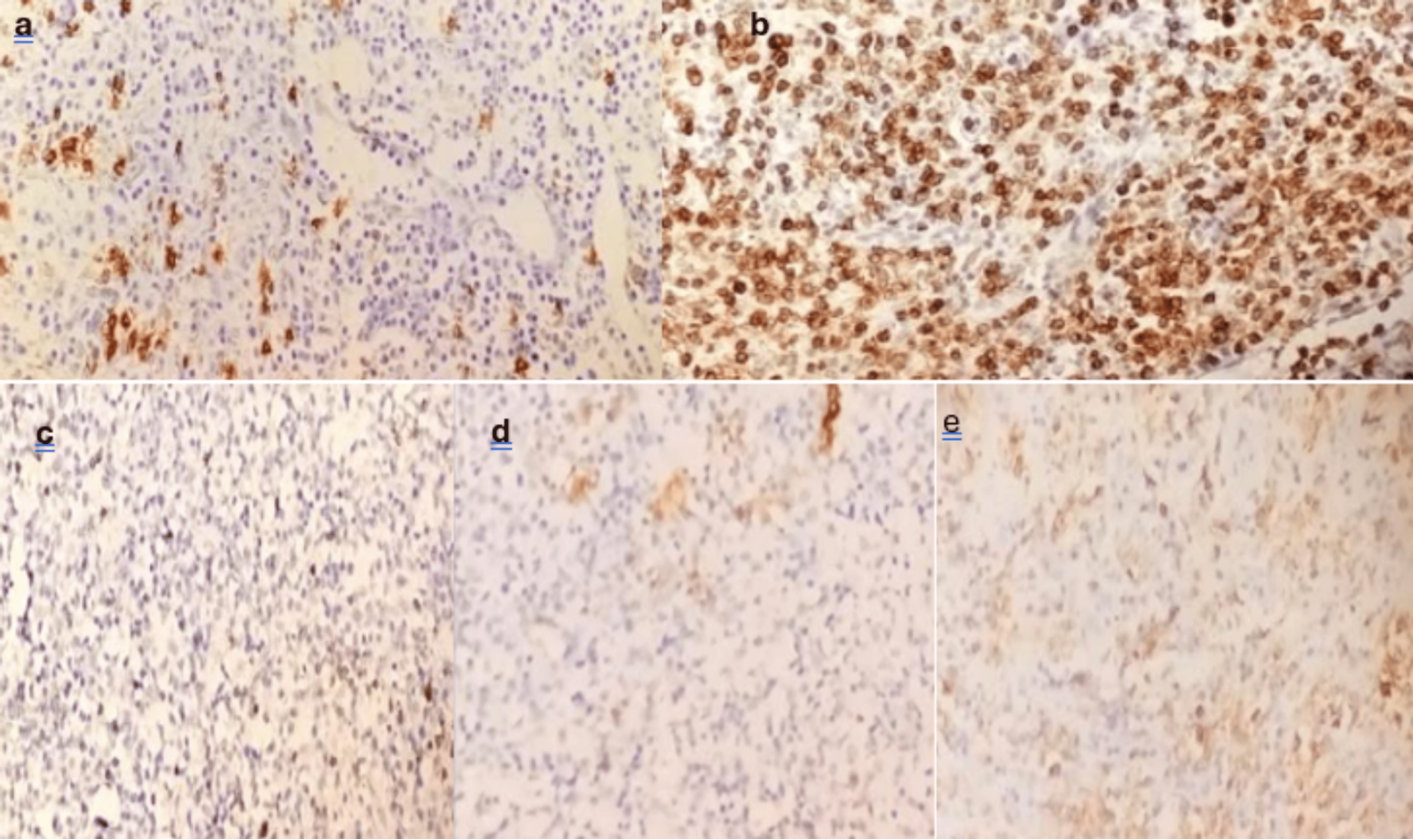
Angioimmunoblastic T-cell lymphoma. (a) Positive staining of immunoblastic cells with anti-CD20 antibodies. (b) Diffuse positive staining of tumor cells by anti-CD3 antibodies. (c) Partial loss of CD7 expression. (d) Positive staining with anti-CD23 antibody, highlighting dendritic follicular cell networks. (e) PD1 is positive in the perifollicular T cells PD-1: programmed cell death protein 1

Immunohistochemical analysis revealed diffuse positive staining of tumor cells with anti-CD3, anti-CD5, anti-BCL6, anti-CD2, anti-CD4, and anti-PD1 antibodies, along with partial loss of CD7 expression. Immunoblastic cells exhibited positive staining with anti-CD20 and anti-CD30 antibodies, while tumor cells showed focal labeling with the anti-CD10 antibody. The anti-CD23 antibody highlighted follicular dendritic cell networks, and histiocytes showed reactive labeling with the anti-CD68 antibody. In contrast, tumor cells did not stain with anti-CD15, anti-BCL2, or anti-CD8 antibodies (Figure 5).

Due to the patient's performance score of 4, an exploratory and therapeutic thoracoscopy was recommended but not performed. The diagnosis of AITL with lymph node, lung, and pleural involvement was confirmed. The patient underwent a prechemotherapy workup and was referred to the hematology department to initiate Cyclophosphamide, doxorubicin (Hydroxydaunorubicin), vincristine (Oncovin), and Prednisone (CHOP)-type chemotherapy.

## Discussion

AITL is the second most common subtype of PTCL, with a widely varying geographical incidence: 16% in the United States, 18% in Asia, and 29% in Europe. However, it accounts for only 1%-2% of all non-Hodgkin's lymphomas (NHLs) [3,4]. The mean age of onset in the main clinical studies is between 57 and 68 years [5-7], and to date, no specific risk factors or etiological agents have been identified for AITL. The condition appears to be more frequent in men than in women, and our patient was within this age group [8].

Initial clinical signs of AITL include lymphadenopathy, general symptoms, hepatosplenomegaly, and/or rash, as well as pleural or peritoneal effusions. Pleural effusion is observed in 9%-37% of cases at the time of diagnosis [7-9]. Pulmonary disorders are rare but may present as pulmonary nodules or diffuse interstitial infiltrates, sometimes with a ground-glass appearance [10,11]. The patient's chest CT scan demonstrated a heterogeneous, triangular consolidation in the left upper lung parenchyma, crossed by an air bronchogram and associated with nodular condensation and bilateral bronchopneumonia, an appearance compatible with a lymphomatous origin.

Biological anomalies most frequently encountered in AITL include polyclonal hypergammaglobulinemia, elevated LDH, and anemia [8,9]. Polyclonal hypergammaglobulinemia is observed in more than half of the cases, while in others, hypogammaglobulinemia or the presence of a monoclonal element may also occur. LDH is elevated in 37%-74% of cases, and anemia is observed in 20%-83%, often resulting from bone marrow invasion. Autoimmune hemolysis is present in 13% of patients [12], along with erythroblastopenia or inflammation. Hypereosinophilia is present in one-third of cases, and thrombocytopenia occurs in around 20% of cases, rarely of peripheral origin (e.g., immunological thrombocytopenic purpura or Evans syndrome) [13,14]. Lymphocytosis, neutrophilic polynucleosis, or a circulating contingent may also be observed.

The first-line treatment for AITL is typically based on CHOP-type: chemotherapy, which includes cyclophosphamide, doxorubicin (Hydroxydaunorubicin), vincristine (Oncovin), and prednisone, or Adriamycin, Cyclophosphamide, Vindesine, Bleomycin, and Prednisone, most often in combination with anti-CD20 antibodies due to the B-lymphoid contingent [7]. Bone marrow autotransplantation, whose relevance and efficacy have been demonstrated by recent studies, is recommended for younger patients. The prognosis for AITL is generally unfavorable, influenced by factors such as disease stage, the patient’s overall health, treatment response, and genetic characteristics. The overall survival rate is approximately 30% at five years [8].

## Conclusions

We have reported an exceptionally rare case of NHL revealed by pleuropneumopathy, which underlines the importance of recognizing this rare association in clinical practice. This is an atypical presentation with a generally poor prognosis compared with isolated non-Hodgkin's malignant lymphomas, highlighting the need for early and accurate diagnosis. The appearance of respiratory symptoms may mask the lymphomatous origin of the disease, making it crucial to consider this possibility before diagnosing pleuropneumopathy.

It is also essential to consider other differential diagnoses, such as tuberculosis, which may present with similar clinical and radiological signs. However, confirmation of tuberculosis relies on bacteriological evidence, which requires thorough investigation. Vigilance when faced with these clinical presentations not only accelerates diagnosis but also improves therapeutic management, reducing the risk of serious complications associated with delayed diagnosis.
